# Application of the thermal time model for different *Typha domingensis* populations

**DOI:** 10.1186/s12870-020-02573-3

**Published:** 2020-08-17

**Authors:** Fanny Mabel Carhuancho León, Pedro Luis Aguado Cortijo, María del Carmen Morató Izquierdo, María Teresa Castellanos Moncho

**Affiliations:** 1 Agroenergy Research Group, Department of Agricultural Production. School of Agricultural, Food and Biosystems Engineering, Universidad Politecnica de Madrid (UPM), Avenue Complutense s/n, 28040 Madrid, Spain; 2 Department of Applied Mathematics. School of Agricultural, Food and Biosystems Engineering, Universidad Politecnica de Madrid (UPM), Avenue Complutense s/n, 28040 Madrid, Spain

**Keywords:** *Typha domingensis*, Thermal time, Seed germination, Green floating filter

## Abstract

**Background:**

Cattail (*Typha domingensis* Pers.) is a perennial emergent plant which is used in Green Floating Filters (GFFs), one of the most innovative systems of wastewater treatment to bioremediate eutrophic waters and produce biomass as biofuel feedstocks. The establishment of cattails in GFFs depends on the seed germination and plant responses under conditions of a new habitat. This study analysed the germination responses of four different populations of cattails through a thermal time model to know their basic parameters of germination and which population would be more adapted to the conditions tested.

**Results:**

Seeds from the Badajoz (**Ba**), Cuenca (**Cu**), Madrid (**Ma**), Seville (**Se**) and Toledo (**To**) populations were exposed to different thermal regimes (constant, and alternating temperatures between 15 and 30 °C) and different darkness treatments (between 0 and 20 days with 24 h dark photoperiod, then exposed to light with 12 h light/dark photoperiod) to determine the parameters of the thermal model from germination levels in each treatment. **To** population was used to validate the thermal time parameters of other populations. Regardless of the other parameters, no germination occurred in total darkness. The mean value of base temperature (T_b_) was 16.4 ± 0.2 °C in all treatments. Optimum temperature (T_o_) values in **Ma** and **Ba** were 25 °C, and those in **Cu** and **Se** were 22.5 °C. The germination response decreased when the temperature approached T_b_ and increased when it was close to T_o_. In comparison to alternating temperatures, constant temperatures had the highest germination response and lowest thermal time (θ_T_(50)). Darkness treatments had a direct relationship with θ_T_(50). The population origin also affected seed germination; **Cu** had the highest values of T_o_ and germination response but had a lower θ_T_(50), which coincides with the lowest mean ambient temperatures.

**Conclusion:**

According to these results, the germination response of cattails was high in all populations under optimal conditions but was affected to a greater or lesser extent depending on thermal regimes, darkness treatments, and populations. The thermal time model allowed us to determine that T_o_ was between 22.5–25 °C and that **Cu** is the best population regarding the germination response under the conditions tested.

## Background

*Typha* is a perennial monocotyledon plant genus that is widely distributed [1] mainly in wetlands, marshlands and other aquatic habitats. *Typha domingensis* Pers*.* (commonly known as “cattail”) is a species of this genus, with an annual growth cycle, that is widely distributed around the world [1]. It is a warm temperate and pantropical species [2]. This species is also often found in the Iberian Peninsula and the Balearic Islands [3]. The reproduction of this species occurs by vegetative spread (rhizomes) and from seed [4, 5]. Seed production is very high due to a single female spadix that can produce more than 600,000 small single-seeded fruits [1]. Although considered an invasive plant, cattail provides different raw materials (for weaving) and the rhizomes serve as food for humans and livestock. In recent years, cattail has been used in wastewater treatments and reclamation of industrial sites due to its great capacity to remove particle matter, nutrients and metals from eutrophic waters [6–8]. For wastewater treatment, this species has been used to form green filters that use different types of biological elements (plants and microorganisms). Among the numerous existing green-filter systems, the innovative systems specially designed for small urban agglomerations are called green floating filters (GFFs) [9]. Different *Typha* species have been used in GFFs, but *T. domingensis* presents an advantage compared to other species: it has the capacity to produce more biomass in deeper water [10, 11] and to quickly form a floating and filtering plant mat that improves the purification of wastewater in a GFF [12]. Different studies have demonstrated that managed cattail in constructed wetlands could provide beneficial ecosystem services [6, 7] and sustainable biomass for biofuel feedstocks [13, 14] and bioproducts [15].

Seed germination is an important biological process in plants. The success or failure of introducing a population into a new local habitat is closely related to its seed germination ability. The dynamics of this process are complex and influenced by genetic changes and/or phenotypic variability in the different plant populations [16, 17]. An example of this complexity is the dormancy mechanism. It is an internal condition of seeds that impedes its germination under otherwise adequate hydric, thermal and gaseous conditions [18, 19]. This mechanism is very rare in seed crops but common in weed populations and is associated with weed dispersibility [20]. Dormancy can be ended by one or more environmental factors, such as temperature, water potential, light, and soil pH. However, different dormancy behaviours are related to environmental factors during seed development [19] and seeds can incorporate the effects of these factors over time [21].

In the case of cattail, germination studies have focused on avoiding its propensity to invade natural ecosystems and cause negative impacts. Previous studies have demonstrated that environmental requirements, such as temperature, light, depth of water, salinity, pH, and O_2_ concentration could influence the seed germination of different species of *Typha* [5, 16, 22–24].

One of the main factors required for seed germination is the temperature [25]. This factor has the greatest effect on dormancy, and on the germination rate in the case of seeds that do not exhibit dormancy [26], and many studies have been implemented using constant temperatures [17, 27, 28] or/and alternating temperatures [29, 30] on seed germination. Light is another environmental factor that is important for releasing seeds from dormancy [25, 31]. Among the environmental requirements related specifically to cattail seed germination, one of the main factors is humidity. Germination of cattail seeds only occurs in wet or flooded environments [5, 32, 33] at low soil water potentials < 1 MPa [34].

Genetic determinants also influence the germination of cattail seeds [35, 36]. Sometimes, the origins of populations determine the germination conditions of the seeds regardless of whether they are of the same species [24, 37]. Moreover, the factors that determine the germination of the mother plant (temperature, light, humidity, and others) must be taken into account [38]. Knowledge of the cattail populations germination capacity can help in the establishment in GFFs or the control of its expansion in different places [39].

There are various tools for the study of seed germination. In recent years, population-based threshold models have been widely used in germination studies. There are two types of models: empirical and mechanistic. The first is used for a specific objective, but the results are more difficult to apply [40, 41]. Mechanistic models, on the other hand, are based on known and experimentally quantified the environmental effects on seed dormancy, imbibition, and germination [41]. These models have been applied, therefore, to explain the most successful seed germination in recent years [29, 42, 43]. The main models developed are the thermal time model, the hydrotime model, and the hydrothermal model, which describe the effects of temperature and/or water potential on the germination rate by applying a linear relationship [42, 44, 45]. These models use biological time, which can be quantified by the extent to which water potential and/or temperature of each seed exceeds thresholds (base), below which germination is not completed [20]. The hydrotime model describes the response germination of seed populations in response to change in water potentials. The thermal time model is based on response germination under variable temperature regimes, and the hydrothermal model is the integration of both temperature and water potential [25, 42, 44]. The use of these models could provide a way to link ecological observation of germination to laboratory studies [20].

The thermal time model allows for the estimation of the width of the thermal range over which seeds of a particular species can germinate. This thermal range can be described by the three cardinal temperatures: optimum temperature (T_o_), base temperature (T_b_), and ceiling temperature (T_c_) [19]. T_o_ is the temperature at which germination is most rapid, while T_b_ and T_c_ are the lowest and highest temperatures at which germination can occur, respectively [25]. Also, the dormancy status of the seeds can influence the thermal range between T_b_ and T_c_ [25]. This model is based on mathematical parameters, such as thermal time (*θ*_*T*_, degree-day/degree-hour), the three cardinal temperatures (T_b_, T_o_ and T_c_, °C), the mean temperature of incubation (T_m_,°C), the germination rate (GR) and the time to germination (t) for a specific germination percentile (g). The *θ*_*T*_ is the daily differences between prevailing temperature and T_b_, which are accumulated to complete germination [30]. GR is considered as the inverse of time to germination for specific germination percentages; moreover, it increases with increasing temperature between T_b_ and T_o_, while it decreases between T_o_ and T_c_ [19]. The thermal time model has been used successfully to predict the occurrence of seed germination under non-water-limiting conditions, thus explaining approximately 80% of the variation in the cumulative percentile [46].

The aims of this work, therefore, was to determine the ecophysiological parameters of seeds germination for different populations of *T. domingensis* from Badajoz (Ba), Cuenca (Cu), Madrid (Ma) and Seville (Se) using thermal time model. These parameters allow us to know the germination behaviour of each population and the influence of environmental parameters, such as level and amplitude of temperatures or light exposure, on its germination responses. This analysis can be useful to know which would be the best population for establishing in a new habitat as well as assessing its ability to expand as a weed in a future scenario with warmer temperatures in the Mediterranean zone because of climate change.

## Results

### Final germination responses in the different treatments

Table 1 shows the germination responses achieved in each treatment. To simplify Table 1, the germination responses to each set of temperatures and darkness treatments within the same population were summarized, and these are shown as lower and upper germination responses for constant (C) and alternating (A) thermal regimes.

**Table 1:** Final germination percentage in *T. domingensis* seeds

Population	Darkness treatments	DT0d		DT3d		DT5d		DT7d		DT10d	
	Thermal regimen	Lower	Upper	Lower	Upper	Lower	Upper	Lower	Upper	Lower	Upper
Ba	C	91 ± 1.9 (17.5)	99 ± 1.3 (25)	59 ± 3.1 (17.5)	99 ± 1.6 (30)	65 ± 3.8 (17.5)	94 ± 2.0 (22.5)	69 ± 5.1 (17.5)	92 ± 1.8 (25)	58 ± 5.3 (17.5)	79 ± 4.7 (22.5)
	A	60 ± 4.5 (15/20)	99 ± 4.1 (20/25)	53 ± 5.2 (15/20)	98 ± 1.7 (15/30)	53 ± 4.5 (15/20)	94 ± 2.6 (15/30)	53 ± 6.1 (15/20)	93 ± 4.4 (15/30)	49 ± 7.6 (15/20)	91 ± 4.1 (15/30)
Cu	C	92 ± 1.2 (17.5)	99 ± 1.7 (22.5)	87 ± 2.7 (17.5)	99 ± 1.1 (22.5)	81 ± 2.9 (17.5)	98 ± 1.3 (22.5)	74 ± 3.6 (17.5)	99 ± 1.8 (22.5)	67 ± 4.2 (17.5)	94 ± 3.9 (22.5)
	A	61 ± 5.5 (15/20)	99 ± 3.2 (15/25)	55 ± 6.9 (15/20)	97 ± 4.6 (20/30)	54 ± 7.7 (15/20)	98 ± 1.3 (15/30)	49 ± 6.2 (15/20)	95 ± 2.7 (20/25)	51 ± 7.1 (15/20)	94 ± 1.5 (15/30)
Ma	C	89 ± 2.2 (17.5)	99 ± 2.1 (25)	72 ± 2.7 (17.5)	98 ± 2.8 (30)	73 ± 3.3 (17.5)	96 ± 2.0 (25)	65 ± 4.5 (17.5)	97 ± 2.2 (30)	48 ± 6.4 (17.5)	86 ± 1.6 (25)
	A	67 ± 6.2 (15/20)	97 ± 3.1 (15/30)	51 ± 4.3 (15/20)	99 ± 2.1 (20/30)	48 ± 6.7 (15/20)	98 ± 3.6 (20/30)	49 ± 7.9 (15/20)	94 ± 3.4 (30)	49 ± 8.4 (15/20)	93 ± 3.4 (15/30)
Se	C	86 ± 3.8 (17.5)	99 ± 4.6 (22.5)	92 ± 5.8 (17.5)	97 ± 4.5 (22.5)	75 ± 5.5 (17.5)	92 ± 4.2 (22.5)	66 ± 6.5 (17.5)	91 ± 4.9 (30)	53 ± 8.2 (17.5)	83 ± 4.4 (22.5)
	A	58 ± 4.5 (15/20)	95 ± 3.0 (15/25)	52 ± 6.8 (15/20)	92 ± 4.3 (15/25)	53 ± 6.6 (15/20)	84 ± 5.44 (15/30)	54 ± 7.2 (15/20)	85 ± 5.2 (25/30)	50 ± 6.3 (15/20)	79 ± 4.9 (15/30)
Validation data (To)	C	74 ± 4.9 (17.5)	99 ± 3.1 (22.5)	62 ± 8.6 (27.5)	92 ± 6.5 (22.5)	62 ± 3.9 (17.5)	90 ± 4.2 (22.5)	56 ± 6.5 (17.5)	96 ± 4.9 (30)	47 ± 8.2 (17.5)	83 ± 4.4 (22.5)
	A	62 ± 5.2 (15/20)	96 ± 4.4 (15/25)	54 ± 7.9 (15/20)	96 ± 3.6 (15/25)	49 ± 8.6 (15/20)	97 ± 4.3 (15/30)	51 ± 9.1 (15/20)	97 ± 4.4 (25/30)	39 ± 9.8 (15/20)	95 ± 3.6 (15/30)

Maximum and minimum values ± SD of final germination percentage achieved according to different populations, thermal regimes, and darkness treatments in cattail seeds. The temperature, at which these percentages were reached, is shown in parentheses on the bottom line

The results of the multifactor analysis of variance carried out with the data mentioned above are shown in Table 2. There were significant differences among the populations (Po), darkness treatments (DT) and thermal regimes (Tr) when each of these factors was analyzed separately at *p* < 0.05; however, the interaction between the two factors (PoxDP, DPxTr, PoxTr) and among the three factors (PoxDPxTr) was not significant (Table 2).

**Table 2:** Multifactor analysis of variance and multiple range tests for different populations, darkness treatments and thermal regimes

Multifactorial ANOVA			Multiple Ranges test								
Factors	Fd	F	Po	n	GR %	DT	N	GR %	Tr	n	GR%
Po	3	22.66^***^	Ba	60	82.2^b^	DT0d	48	91.4^a^	17.5_0	20	74.0^d^
DT	4	30.77^***^	Cu	60	88.2^a^	DT3d	48	87.6^b^	17.5_5	20	54.0^e^
Tr	5	51.45^***^	Ma	60	85.9^a^	DT5d	48	82.3^c^	20.0_0	20	83.0^bc^
PoxDT	12	0.08	Se	60	76.6^c^	DT7d	48	79.9^c^	20.0_10	20	82.0^bc^
PoxTr	9	0.68				DT10d	48	75.1^d^	22.5_0	20	92.0^a^
DTxTr	12	0.73							22.5_5	20	89.0^ab^
PoxDTxTr	36	0.15							22.5_15	20	93.0^a^
									25.0_0	20	92.0^a^
									25.0_10	20	87.0^abc^
									27.5_0	20	88.0^abc^
									27.5_5	20	82.0^c^
									30.0_0	20	88.0^abc^

*Po* Populations; *DT* Darkness treatments; *Tr* Thermal Regimes (T_m__ΔT)

Significant codes: *** 0.001, ** 0.01, * 0.05. Different letters represent statistically significant differences between treatment of each population according to LSD test (p < 0,05)

No germination was obtained in DT20d treatment (20 days with 24 h darkness photoperiod), so the data from these treatments have not been included in the study. These results suggest that the dormancy of cattail seeds was not interrupted independently of thermal treatment or population. In others darkness treatments, different germination responses were reached according to the influence of the factors studied (Table 1), and there was an inverse relationship (Cor. Coeff: − 0.99, p < 0,05) between the number of dark days and the germination response within the same population and thermal regimes (Table 2). This difference was observed between the DT0d versus DT10d. Regarding the origin of the seeds, there were no significant differences between **Cu** and **Ma**, but differences did exist between **Ba** and **Se**, and those two together were significantly different from **Cu** and **Ma** together. (Table 2). **Cu** and **Ma** had the highest values, while **Se** had the lowest germination responses.

### Thermal model

**Fig. 1:**
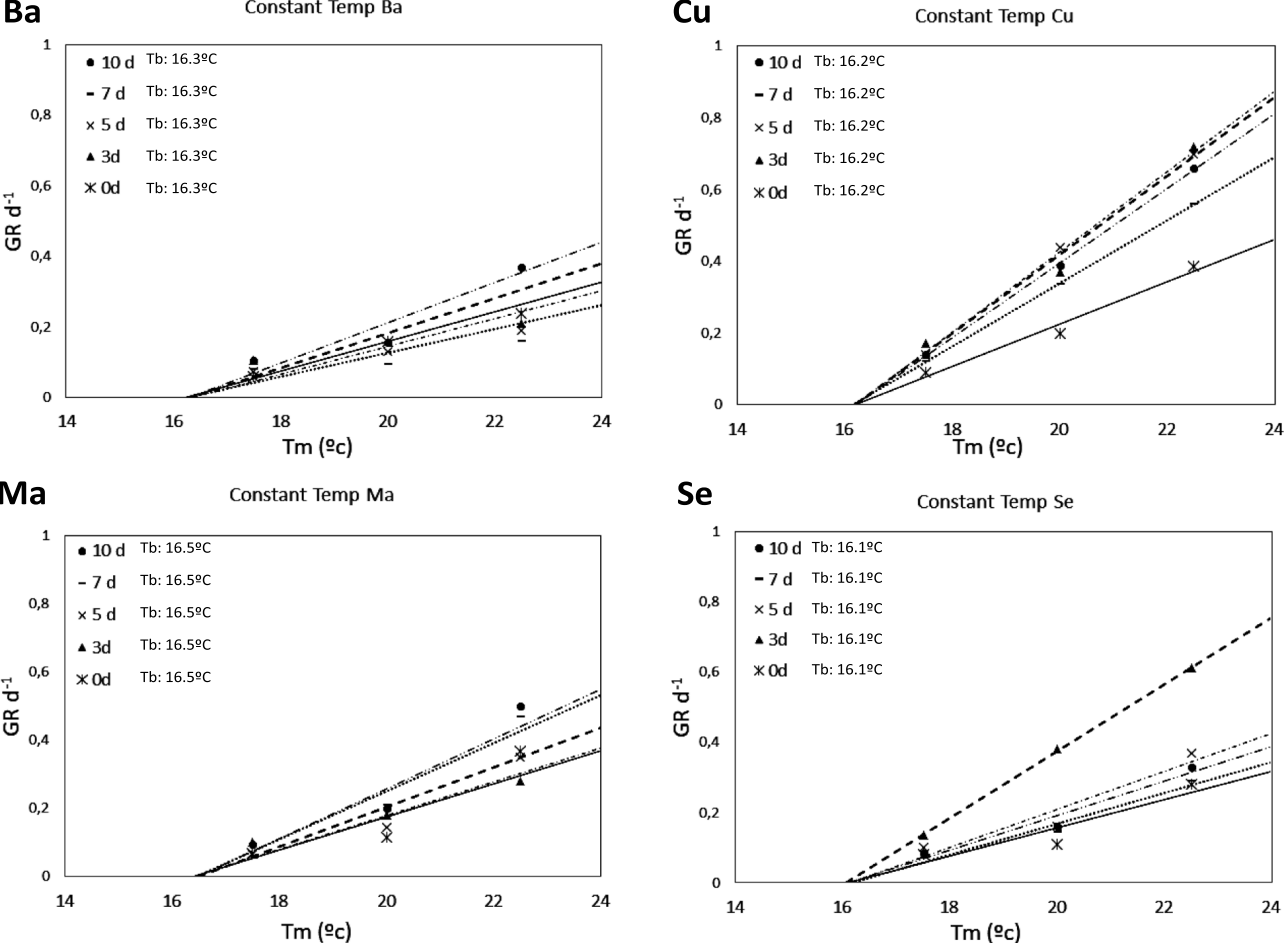
Relationship between GR _(50)_ and T_m_ of cattail seeds from different populations with ΔT = 0 °C and different darkness treatments

**Fig. 2:**
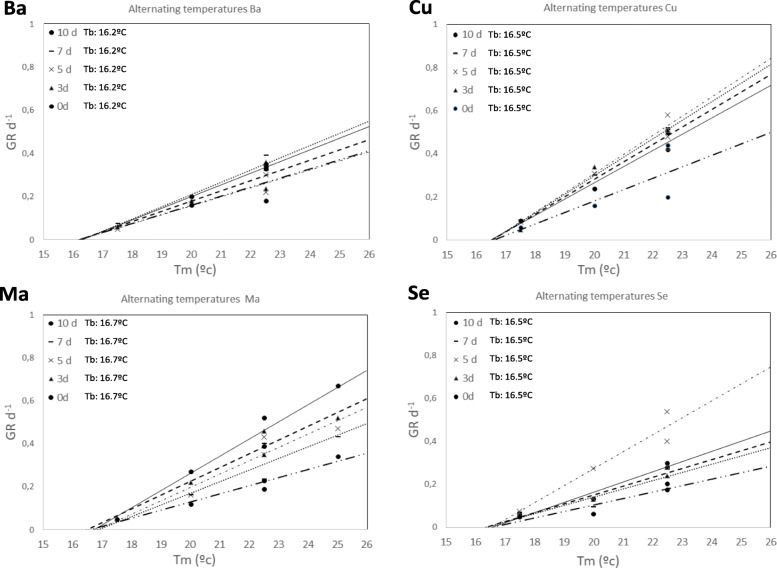
Relationship between GR _(50)_ and T_m_ of cattail seeds from different populations with ΔT ≥ 0 °C and different darkness treatments

The differences in the thermal regimes depended on the mean temperature (T_m_) of each regime. In Figs. 1 and 2, the relationships are shown between GR_50_ and cattail seeds of distinct populations, thermal regimes and darkness treatments. The mean value of T_b_ was 16.4 ± 0.2 °C with a minimum of 16.1 °C and a maximum of 16.7 °C. This value explained why no germination occurred in the thermal treatments lower than 17.5 °C and the lowest values of the germination responses occurred in the thermal treatments closest to T_b_ (Table 2).

**Fig. 3:**
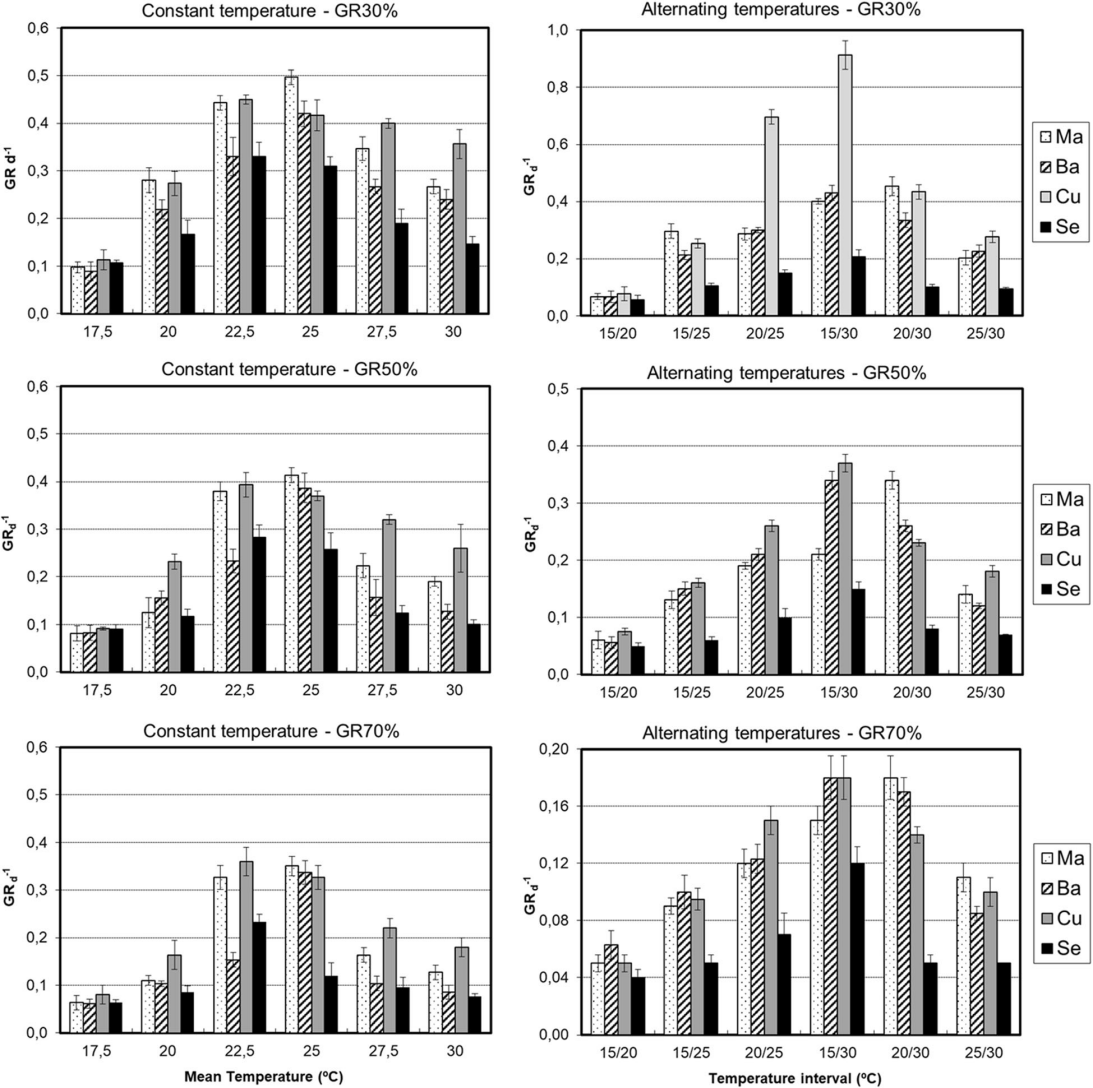
Relationship between GR_(g)_ and T_m_ in treatments within constant and alternating temperatures and darkness treatment = DT0d

In Fig. 3, the relationships are shown between GR of different percentiles (30, 50 and 70%) and T_m_ of different populations with constant and alternating temperatures and treatments without 24 h dark photoperiod (DT0d). In thermal regimes within constant temperatures (Fig. 3a), both **Ma** and **Ba** had a T_o_ = 25 °C in the three percentiles of GR, while **Cu** and **Se** had a T_o_ = 22.5 °C. In the regimes with alternating temperatures (Fig. 3b), only **Ma** had a T_o_ = 25 °C, while the remaining populations had T_o=_22.5 °C. The T_o_ was 22.5 °C, but the optimum temperature difference (ΔT_o_) =2.5 °C was found in the **Ma** population with constant and alternating temperatures and the **Ba** population at constant temperatures (Fig. 3).

The thermal regimes with T_m_ and the temperature difference (ΔT) =0 °C close to T_b_ achieved the lowest germination responses in all the treatments, while the thermal regimes at temperatures close to T_o_ showed the highest responses. The highest germination responses were observed in treatments with ΔT = 0 °C. The germination responses in the thermal regimes with the same T_m_ but with ΔT = 5 °C/10 °C were lower than ΔT = 0 °C, while the germination in thermal regimes with ΔT = 15 °C was much closer to the germination with ΔT = 0 °C (Table 2).

The values of T_o_ with different darkness periods were the same as those at DT0d (Table 3). The highest values of GR_50_ (0.34) were reached within constant regimes close to T_o_ (22.5/25 °C) in all populations (Fig. 3a). In alternating regimes (Fig. 3b), these values are all approximately 0.23 in T_o_ = 25 °C. ANOVA of alternating temperatures indicated significant differences between thermal treatments (F _(5, 61)_=9.17, p < 0,001); therefore, the thermal treatments 15/30 °C and 20/25 °C showed different values of three percentiles of GR (30,50 and 70%). Although they had the same mean temperature (T_m_), this difference could be related to the different ranges utilized in alternating temperatures (15 and 5 °C, respectively).

**Table 3:** Parameters of the thermal models for cattail seeds with constant (A) and alternating (B) temperatures

A. Thermal regimes with constant temperatures							B. Thermal regimes with alternating temperatures						
Code	T_b_ °C	T_o_ °C	Log(θ_T_(50)) (log°d)	ơθ_T_ (log°d)	θ_T_(50) (°d)	θ_T_(50) (°h)	Code	T_b_ °C	T_o_ °C	Log(θ_T_(50)) (log°d)	ơθT (log°d)	θ_T_(50) (°d)	θ_T_(50) (°h)
BaCDT0d	16 ± 0.3	25	2.72	0.5	15.33	368	BaADT0d	16 ± 0.2	22.5	2.81	0.3	16.61	399
BaCDT3d	16 ± 0.3	25	2.81	0.5	16.61	399	BaADT3d	16 ± 0.2	22.5	2.94	0.3	18.02	454
BaCDT5d	16 ± 0.3	25	2.87	0.5	17.64	423	BaADT5d	16 ± 0.2	22.5	3.00	0.3	20.09	482
BaCDT7d	16 ± 0.3	25	2.96	0.5	19.21	461	BaADT7d	16 ± 0.2	22.5	3.06	0.3	21.33	512
BaCDT10d	16 ± 0.3	25	3.06	0.5	21.27	510	BaADT10d	16 ± 0.2	22.5	3.12	0.3	22.65	544
CuCDT0d	16 ± 0.2	22.5	2.41	0.4	11.13	267	CuADT0d	16 ± 0.5	22.5	2.70	0.4	14.88	357
CuCDT3d	16 ± 0.2	22.5	2.50	0.4	12.18	292	CuADT3d	16 ± 0.5	22.5	2.73	0.4	15.45	378
CuCDT5d	16 ± 0.2	22.5	2.63	0.4	13.87	333	CuADT5d	16 ± 0.5	22.5	2.79	0.4	16.28	391
CuCDT7d	16 ± 0.2	22.5	2.71	0.4	15.03	361	CuADT7d	16 ± 0.5	22.5	2.87	0.4	17.58	422
CuCDT10d	16 ± 0.2	22.5	2.84	0.4	17.11	411	CuADT10d	16 ± 0.5	22.5	2.90	0.4	18.23	438
MaCDT0d	16 ± 0.5	25	2.57	0.5	13.06	314	MaADT0d	16 ± 0.7	25	2.76	0.4	15.80	379
MaCDT3d	16 ± 0.5	25	2.70	0.5	14.92	358	MaADT3d	16 ± 0.7	25	2.80	0.4	16.44	395
MaCDT5d	16 ± 0.5	25	2.80	0.5	16.44	395	MaADT5d	16 ± 0.7	25	2.91	0.4	18.30	419
MaCDT7d	16 ± 0.5	25	2.90	0.5	18.17	436	MaADT7d	16 ± 0.7	25	3.00	0.4	20.09	482
MaCDT10d	16 ± 0.5	25	3.01	0.5	20.29	487	MaADT10d	16 ± 0.7	25	3.03	0.4	20.78	499
SeCDT0d	16 ± 0.1	22.5	2.75	0.4	15.70	377	SeADT0d	16 ± 0.5	22.5	2.98	0.4	19.69	473
SeCDT3d	16 ± 0.1	22.5	2.80	0.4	16.44	395	SeADT3d	16 ± 0.5	22.5	3.02	0.4	20.49	498
SeCDT5d	16 ± 0.1	22.5	2.89	0.4	17.99	432	SeADT5d	16 ± 0.5	22.5	3.11	0.4	22.42	538
SeCDT7d	16 ± 0.1	22.5	3.08	0.4	21.76	522	SeADT7d	16 ± 0.5	22.5	3.21	0.4	24.78	595
SeCDT10d	16 ± 0.1	22.5	3.16	0.4	23.57	566	SeADT10d	16 ± 0.5	22.5	3.24	0.4.	25.53	613

T_b_ = Base temperature, T_o_ = Optimum temperature; log(θ_T_(50)) = log thermal time 50% germination in °d; σθ_T_ = standard deviation of the log thermal time distribution within the seed population in °d; θ_T_(50)= thermal time 50% germination in °d and °h, respectively

**Fig. 4:**
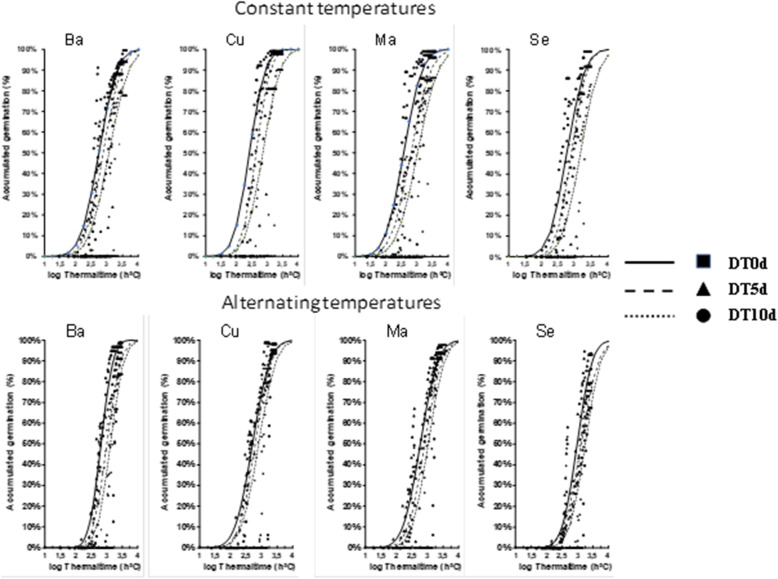
Relationship between accumulated germination and log(Ɵ_T_(g)) in different populations with temperatures and darkness treatments

In Fig. 4, the relationship between accumulated germination and log(θ_T_ (50)) in the different thermal regimes (C and A), populations and darkness treatments are shown. The curves of the models within the same population were close but not equal, and slight differences were observed between different darkness treatments and between thermal regimes with constant versus alternating temperatures. The curves of the regimes with alternating temperatures shift to the right when compared with those of constant temperatures. There were similar values of ơθ_T_ (standard deviation of the log thermal time) in all treatments (Table 3) which means that final germination (50%) was reached in all treatments.

The treatments of the same population and darkness treatments in thermal regimes with constant temperatures showed lower values of thermal time (Table 3) in comparison with regimes of alternating temperatures. The thermal time was influenced by the darkness treatments because, for all populations, and all temperature levels and temperature amplitudes, it was seen to increase the more days the seeds were kept in darkness(Cor. Coeff: − 0.99, p < 0,05). According to these results, there was a relationship between log(θ_T_ (50)) and the set of darkness treatments (Table 4) characterized by an R^2^ > 0.90.

**Table 4:** Relationship between log(θ_T_(50)) and darkness treatments (DT) in treatments with the same population and amplitude of temperature regimes

Code	A	b	R^2^
BaC	2.71	0.034	0.993
BaA	2.83	0.031	0.965
CuC	2.39	0.044	0.986
CuA	2.69	0.022	0.927
MaC	2.57	0.045	0.996
MaA	2.75	0.030	0.910
SeC	2.71	0.045	0.908
SeA	2.96	0.029	0.938

Log(θ_T_(50)) = a + b* (DT)

There were also differences between θ_T_ (50) according to populations (Table 3). The lowest θ_T_ (50) time (Table 3) corresponded to **Cu,** which coincided with the lowest yearly maximum, mean and minimum ambient temperatures (Table 5). **Ma** had θ_T_ (50) higher than that of **Cu** with ambient temperatures slightly higher than those in **Cu**, while **Se** and **Ba** had the highest θ_T_(g) (Table 3) and the highest ambient temperatures (Table 5).

**Table 5:** Codes, population name, geographic coordinates and temperatures of five locations where seeds were collected

Code	Population	Latitude	Longitude	*MATo*_*ax*_ *(°C)*	*MATo* *(°C)*	*MATo*_*in*_ *(°C)*
**Ba**	Puebla de Alcocer, Badajoz	38°59′N	5°15′W	23.8	17.1	10.3
**Cu**	Olmedilla del campo, Cuenca	40°03′N	2°42′W	19.3	13.1	6.9
**Ma**	Ciudad Universitaria, Madrid	40°26′N	3°44′W	19.9	15.0	10.1
**Se**	Lantejuela, Seville	37°21′N	5°13′W	25.4	19.2	13.0
**To**	Seseña, Toledo	40°04′N	3°37′W	22.1	15.8	9.5

MATo_ax_ (yearly mean maximum temperature); MATo (yearly mean temperature) and MATo_in_ (yearly mean minimum temperature). Historical data obtained from The State Meteorological Agency of Spain [47]

The results of the evaluation of the thermal time models are shown in Table 6. The coefficient of determination (R^2^) was the concordance between Log(θ_T_(g)) of each model (expected values in each model) and Log(θ_T_(g))_**To**_ (observed values). Results of R^2^ varied depending on thermal regimes and darkness treatments. A good concordance was found in all treatments with R^2^ values greater than 0.77. The highest values of R^2^ (≥ 0.90) corresponded to treatments without 24 h dark photoperiod (DT0d) in all populations and thermal regimes except **Ma** population (0.87 and 0.85 respectively), which implied a strong coincidence between expected and obtained values. R^2^ values decreased in treatments with 24 h dark photoperiod (DT3d, DT5d, DT7d and DT10d).

**Table 6:** Evaluation of the thermal time models. Difference between log(*θ*_*T*_(g)) (expected values) versus log(*θ*_*T*_(g))_To_ (observed values)

CODE	R^**2**^	Std Err	CODE	R^**2**^	Std Err
BaCDT0d	0.91	0.13	BaADT0d	0.91	0.09
BaCDT3d	0.84	0.12	BaADT3d	0.86	0.10
BaCDT5d	0.81	0.13	BaADT5d	0.84	0.11
BaCDT7d	0.79	0.13	BaADT7d	0.83	0.11
BaCDT10d	0.80	0.22	BaADT10d	0.82	0.12
CuCDT0d	0.94	0.06	CuADT0d	0.90	0.14
CuCDT3d	0.82	0.11	CuADT3d	0.82	0.14
CuCDT5d	0.81	0.08	CuADT5d	0.81	0.13
CuCDT7d	0.77	0.11	CuADT7d	0.81	0.13
CuCDPT10d	0.77	0.13	CuADPT10d	0.80	0.13
MaCDT0d	0.87	0.16	MaADT0d	0.85	0.12
MaCDT3d	0.83	0.14	MaADT3d	0.82	0.12
MaCDT5d	0.81	0.18	MaADT5d	0.78	0.18
MaCDT7d	0.81	0.18	MaADT7d	0.80	0.27
MaCDT10d	0.81	0.15	MaADT10d	0.80	0.16
SeCDT0d	0.94	0.09	SeADT0d	0.91	0.10
SeCDT3d	0.83	0.05	SeADT3d	0.85	0.08
SeCDT5d	0.82	0.11	SeADT5d	0.79	0.23
SeCDT7d	0.78	0.18	SeADT7d	0.79	0.27
SeCDT10d	0.78	0.11	SeADT10d	0.78	0.23

## Discussion

The successful establishment of a plant species in a location is closely related to the rapidity of germination. Different genotype and/or environmental factors can affect this process by increasing or decreasing this rate. Amongst the environmental factors, light is one that does not prevent the germination of seeds, if it acts as a signal [25] to cause a change in the germination rate and final germination [31] and, therefore, in thermal time parameters. This factor is one of the main determinants of the accumulation of a persistent seed bank of numerous weeds in the soil [48], and it is necessary for the germination of many species [31] mainly of plants with small seeds [31, 48] because large seeds can emerge from a much greater depth than light can penetrate [49]. Exposure time to light may be short, less than a minute, or long. Short exposure time is more commonly effective with small weed seeds, such as cattail seeds, than with large weeds [31].

Light exposure influences the germination of different *Typha* species [5, 50]. In this work, no germination was obtained in DT20d treatments and a delay in the germination was observed in treatments with a 24 h dark photoperiod (DT3d to DT10d). This effect may be explained by the development of a secondary dormancy related to phytochrome activation/deactivation processes which occur through the stimulus of light on cattail seeds. Phytochromes are the principal mechanism triggering germination of *Typha* because they participate in breaking the dormancy [22, 25]. These pigments have two mutually photoconvertible forms: Pfr (considered the active form for seed germination) and Pr (considered the inactive form) [25, 49]. Pfr is established during the formation of the seed in the mother plant; however, this phytochrome form can reconvert to Pr in darkness [18, 31]. In these circumstances, the secondary dormancy does not break, and a period is needed to reconvert the phytochrome to its active form (Pfr) [51, 52]. This secondary dormancy can explain the results in darkness treatments. For example, in the darkness treatments (DT3d to DT10d), thermal time increases as the number of days in darkness increases (Table 4). In the case of DT20d treatments, no germination was measured after 20 days in darkness. These cattail seeds, although they absorbed water and began to swell, did not break their coatings to allow germination. This may explain the death of every seed after 20 days in darkness or the delay produced by secondary dormancy. We support this second option but, since no subsequent germination data was collected, a further study would be necessary to determine it.

Treatments of the same population had an increase in θ_T_ (50) as the number of days in darkness increased. There was a relationship between log(θ_T_ (50)) and darkness treatments (R^2^ > 0.90) (Table 4). Initially, a linear increase in thermal time was expected as the number of days in darkness increased. Indeed, there was an increase, but it was not proportional; for example, in the case of the population of **Cu** with constant temperatures, θ_T_ (50) at PT0d was 267 °h, and the value corresponding to DT10d was 411 °h. This means a 50% increase in thermal time, not a 100% increase as expected. This modification would indicate that *T. domingensis* seeds accumulate hours of temperature and that when receiving light, the dormancy is broken by the activation of Prf and the germination response occurs more quickly than expected. Darkness treatments, such as DT3d, DT5d, DT7d and DT10d, had lower seed germination than treatments without 24 h dark photoperiod (DT0d). These results indicate that long days of darkness may decrease the light sensitivity of *T. domingensis*. Dormancy broken in the presence of light and the influence of phytochrome has been studied and is common in small seeds, such as cattails [22, 52]. A buried environment is associated with darkness and cattail seeds do not germinate in darkness at any temperature; hence, buried seeds of *T. domingensis* could be a control method for the establishment in aquatic ecosystems. Darkness is also related to the depth of water [25]; so the establishment of cattails in the GFF system may be successful if seeds are sowed above the soil submerged in water or on a floating structure of this system.

Although water depth was not a factor in this study, it is another factor that is related to the amount of light and the ease of germination of cattail seeds. The depth used was enough to saturate the paper and seed (< 0.4 cm) due to the fact that germination in cattail seeds is greater and faster in aquatic conditions [2, 24, 33]. Some authors have stated that flooded areas increase the germination of *Typha* species, and this increase in germination has a direct relation to depth [17, 53]. This feature may be caused by a decrease in the level of oxygen, rather than by the lower intensity of light in these situations [33]. Other studies, however, show no relationship between the germination rate and depth [34, 54]. The limit of the depth of germination in *Typha* species in clear water is around 40 cm [2] or 1 cm in sediment [55]. There is an extreme case where cattail seeds germinated under 80 cm water (and survived 8 weeks) [56].

The germination response in plants of different origins could also be different [49]. Differences related to the origin of a population are frequent in numerous species of plants, whether crops [57] or weeds [27, 58]. Successfully colonizing a new location is related to the greater adaptive capacity of these populations to harsher environmental conditions compared to other populations [59], thus allowing these populations to have greater flexibility and adaptability to different locations or future climate change scenarios [17].

**Fig. 5:**
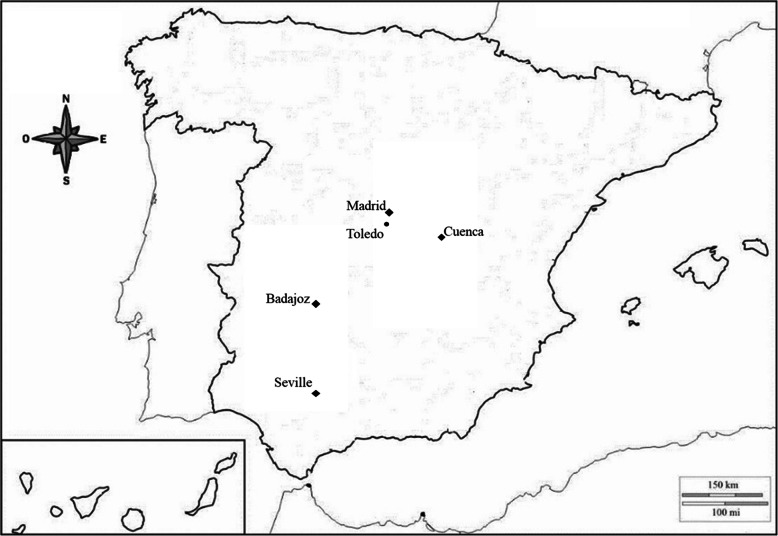
Origin of the populations of *T. domingensis* used in this work. Puebla de Alcocer, Badajoz (**Ba**); Olmedilla del Campo, Cuenca (**Cu**); Lantejuela, Seville (**Se**), the macrophytes nursery of GA, Madrid (**Ma**) and Seseña (**To**). **To** population was used to validate the thermal time parameters

Cattail seeds were grouped into northern (**Cu** and **Ma**) and southern (**Ba** and **Se**) populations (Fig. 5). Mean temperatures of germination within each group were similar, but there were differences between the groups. The northern populations have lower values than the southern populations (Table 5). The results of the thermal time study also show differences between northern and southern populations. In treatments with the same temperature and darkness periods, the northern population presented lower values of thermal time and a higher germination response than the southern populations (Tables 2 and 3).

These differences among populations are consistent with the results of other studies carried out with *Typha latifolia* L. in fifteen European populations [17] and USA populations [60]; in both studies, in comparison to northern populations, southern populations germinated at a lower temperature. However, in our study, the opposite scenario occurred. Before providing conclusions, some points concerning these studies must be clarified. For example, *T. domingensis* is a species more adapted to warmer areas compared to *T. latifolia*. In the European study, only two Mediterranean populations were used, and both populations germinated more rapidly than northern populations; the distances between the origins of the populations were greater than those in our study. Some authors mention that other factors, such as temperature or nutrient supply, are more important than the origin of the seeds in the case of neighbouring populations [17].

In this study, the estimated mean T_b_ was 16.4 °C and no differences greater than 0.6 °C were observed regardless of origin, darkness treatments, or level or amplitude of temperatures. We could have considered that T_b_ was constant; however, other studies with crops [46] or weeds [61] estimated different T_b_ values for the different amplitudes of temperatures. There were significant differences in the germination responses both in terms of the level and amplitude of temperatures (Table 2). In comparison to treatments with other T_m_, treatments with T_m_ close to T_b_ achieved a lower germination response in all treatments (Table 2). No data were found for the calculated T_b_ for *Typha* species, but the estimated values of T_b_ for cattail seeds in this study were very similar to those obtained in other studies with summer weeds [29, 62]. Steinmaus (2000) established a relation between the slope of the line used to estimate T_b_ and germination rate; this rate will be greater with a higher slope. In our study, higher slopes occurred in **Cu** in thermal regimes with both constant and alternating temperatures and coincided with the lower θ_T_ (50) of all populations studied (Figs. 1 and 2).

Differences in T_o_ were obtained in the results of the multifactor analysis, mainly between the northern (**Cu** and **Ma**) and southern populations (**Ba** and **Se**) (Table 4). This difference in T_o_ is comparable with the results of other studies with different populations of weeds or with *T. latifolia* [17, 22, 23]. The T_o_ for the Swedish populations of *T. latifolia* was approximately 20 °C [23] or 10/30 °C with alternating temperatures in Italian populations [22]. Australian populations of the *Typha* genus germinate readily at high temperatures and decline when the T_m_ is lower than 20 °C [63].

Table 7 shows the results from different studies of the seed germination of *T. latifolia* and *T. domingensis*. There are few studies on the seed germination of *T. domingensis*. Lorenzen et al. (2000) stated that a T_o_ of 30 °C and 25/10 °C occurred in south-eastern American populations of *T. domingensis* at constant and alternating temperatures, respectively. These T_o_ values are distinct from those obtained in our study (22.5–25 °C), but there are other studies with T_o_ values very similar to those obtained in this work (Table 7). These results showed different T_o_ values according to the places of origin of the seed and were closely related to climatic conditions at each location [17]. Some conditions, such as the temperature of the mother plants [38, 65], may determine the germination of populations, regardless of whether the seeds were of the same species [24, 37].

**Table 7:** Optimal temperature in *T. domingensis* and *T. latifolia* in different populations from various studies

Plant species	Reference	C	A	Seed location
*Typha domingensis*	This study	22.5; 25 °C		Spain
	Lorenzen et al. (2000) [5]	30 °C	25/10 °C	Florida, U.S.
	Royal Botanic Gardens (2002) [64]	20 °C		Wakehurst, England
*Typha latifolia*	Sifton H.B (1959) [50]	30 °C	20/30 °C	Ontario, Canada
	Bonnewell, V. et al. (1983) [33]	35 °C		Minnesota, U.S.
	Lombardi, T et al. (1997) [22]		20/30 °C	Pisa, Italy
	Ekstam and Forseby (1999) [23]	20 °C		Linköping, Sweden
	Heinz, S (2011)	25 °C	10/25 °C	Germany
	Meng, H. et al. (2016) [24]		25/15 °C	Northeast of China.

*C* constant temperature. *A* alternating temperature

In the *Typha* genus, temperature and amplitude were shown to be factors related to germination [23]. The favourable effect of alternating temperatures on the germination response is well known in different weeds [22] because the effect enables a seed to understand when it is buried and to inhibit germination. In nature, seeds of the cattail are usually submerged. In this situation, fluctuations in the ambient temperature are rare; therefore, an increase in this fluctuation could indicate that seeds have reached land and germination could be stimulated. In this study, both thermal factors (level and fluctuation in temperatures) influenced the final germination of cattail seeds. In the treatments within the same population and in the darkness treatment, there was a greater germination response as the temperature approached T_o_ from values close to T_b_, causing the existence of significant differences depending on the temperature level (Tables 1 and 2). An increase in the germination response is obtained with higher temperatures up to T_o_; above this value, germination begins to decrease. The same results occur in other studies with *Typha* [17, 22, 23, 33] and weeds [27, 29].

The use of different amplitudes of temperature is related to the loss of dormancy in weeds [29, 66] or crops such as lentil [30]. In the case of cattail seeds, the loss of dormancy is related to changes in germination responses. Treatments with ΔT = 0 °C and 15 °C had a higher germination response than those with ΔT = 5 °C and 10 °C (Table 1), so these last two amplitudes of temperature negatively affect germination. However, in studies with *T. latifolia*, treatments with constant temperature regimes (ΔT = 0 °C) achieved a lower germination response than alternating regimes (ΔT > 0 °C) [17]. On the other hand, θ_T_ (50) corresponds to treatments of the same population, and ΔT = 0 °C is lower than treatments with ΔT ≥ 0 °C (Fig. 4), in contrast to *Solanum physalifolium* [29] whose thermal time is considerably reduced in an alternating regime (Table 3). These data are consistent with the germination rate (Fig. 3), in which treatments with alternating temperatures reach lower values than those corresponding to constant temperatures. According to these results, the best season to germinate *T. domingensis* would be late spring because these seasons have a temperature regime of approximately ΔT = 15 °C under natural conditions in the five locations where seeds were collected [47].

The thermal time value of different populations of cattail seeds (Table 3) was substantially lower than that of other weeds such as different species of *Solanum* [20, 22] or tropical species such as *Pennisetum typhoydes* [45, 67]. This indicates a rapid germination response compared with those of other plant species. There were also differences between populations, with **Cu** being the one with the lowest thermal time, both in ΔT = 0 and ΔT > 0 treatments. Although **Cu** and **Ma** obtained similar germination values (Table 2), θ_T_(50) was the highest in **Ma**.

Therefore, **Cu** could be the population that presents the most vigour during this process because this population had the fastest germination under the conditions tested. The final germination percentages were very similar in all populations. It would be necessary to carry out new tests to determine whether the development in other stages of plant growth would also be fast in this population.

In comparison to other species of the genus, such as *T. angustifolia*, *T. domingensis* is a plant species more adapted to warm temperatures. In Spain, it has been observed that *T. domingensis* has been colonizing places where *T. angustifolia* once stood [3]. If this capacity occurs with an increase in temperatures due to climate change, then it is possible to consider that *T. domingensis* might increase its expansion to the detriment of other *Typha* species such as *T. angustifolia.*

According to the evaluation of the models developed in this work (Table 6), there were some differences between the results that were related to thermal regimes and darkness treatments. The greatest coincidences are in the models developed with constant and alternating thermal regimes and DT0d treatments with R^2^ mean values ≥0.9, except for the population of **Ma** that present values slightly below 0.9 in both thermal regimes. The best coincidence was between expected and observed values in constant regimes and treatments without 24 h dark photoperiod (DT0d). Darkness treatments affect the coincidence between expected and observed values, the R^2^ decreased to mean values between 0.84 to 0.78. In the DT3d treatments, R^2^ values were greater than the other darkness treatments. In the remaining darkness treatments (DT5d, DT7d and DT10d), R^2^ values were similar (Table 6). **Ba** and **Ma** populations show both the most and least coincidental values in all treatments, respectively, but the differences were small among all populations.

This work shows that environmental factors and the origin of populations affect the germination responses of cattail seed; moreover, how these parameters could be used to develop models that predicting seed behaviour in a new habitat.

## Conclusions

The thermal time model for the different populations of *T. domingensis* allows an understanding of the germination response of each population established in a new habitat, such as a GFF system. The germination response of *T. domingensis* was affected by thermal regimes, darkness treatments, and populations.

Among the different populations of *T. domingensis*, T_o_ in **Ma** and **Ba** were 25 °C, and those in **Cu** and **Se** were 22.5 °C. However, the T_b_ was the same in the four populations (mean 16.4 ± 0.2 °C). Therefore, the best population can tolerate a vast range of temperatures in a new habitat was **Cu** due to this population had the highest germination rates and the highest germination percentage. If growth chambers are used to proceed with the germination of *T. domingensis* seeds, then the most appropriate temperature treatment will be a constant temperature of 22.5 °C. Under natural conditions, the best time for seed germination occurs when there is a temperature regime of approximately 15 °C in the Mediterranean zone such as Spain, which mainly occurs in the late spring. Values of T_m_ near T_o_ and ΔT values approximately to 15 °C are common in the four populations; hence, these values show that *T. domingensis* could readily germinate in a new habitat and expand as a weed if an increase in the mean temperature occurs.

## Methods

### Plant material

The plant material used for this study was obtained and subsequently identified by experts of the Botany Unit of the Department of Agrarian Production (UPM). The Botanical key used was: Flora Iberica, Vol. XVIII, Gen. Typha [3]. *T. domingensis* is a species widely distributed throughout Spain. For this reason, no specimens were taken to be included in any Herbarium. According to the International Union for Conservation of Nature and Natural Resources (IUCN) Red List Categories, *T. domingensis* does not qualify as critically endangered, endangered, vulnerable or near threatened [68] so permissions were not necessary to collect samples.

The seed material for this study came from natural *T. domingensis* stands at five different locations in Spain (Fig. 5). For the thermal study, mature spadices were collected from Puebla de Alcocer (**Ba**), Olmedilla del Campo (**Cu**), and Lantejuela (**Se**). The plants of these populations were located in naturally flooded areas (ponds, lagoons, and marshes) and in this study they are represented by **Ba**, **Cu,** and **Se,** for plant populations from Badajoz, Cuenca and Seville, respectively (Table 5). **Ma** location was the fourth population. The seeds of this population were obtained from a macrophyte nursery in the experimental fields of GA, Madrid, whose initial source was the Manzanares River, which is very close to these facilities. The fifth population was collected in Seseña, Toledo (**To**). This population was used to evaluate the results of the thermal models obtained for the other populations. According to the classification of Köppen-Geiger, the five locations are classified as having temperate climates with dry and hot summers. The geographic coordinates and temperatures of the different locations are shown in Table 5 [47].

Mature spadices of these populations were collected from plants grouped into the pure mass of cattails between late summer and early autumn of 2017. These spadices were obtained from various *T. domingensis* plants (7–12 plants per population) that grew in the same physical location. The seeds of each spadix were mixed. In the laboratory, the seeds were removed from these spadices by agitating the fruits in water. Only seeds settling to the bottom of the container were selected as viable seeds for the germination test. Then, the selected seeds were dried on filter paper and stored in a refrigerator (at 5 °C) until they were used in the germination test in the following year.

Previous experiments had been carried out to verify that most of the seeds were viable. Shortly before the experiment, the seeds were treated with 1% sodium hypochlorite to prevent infection during the assay [69], washed with sterile distilled water to eliminate any residue and dried rapidly at room temperature.

### Germination tests

Germination tests were carried out in three identical growth chambers with two photoperiodic regimes: (i) 24 h darkness, the dishes were covered with aluminium foil and placed in black plastic bags to prevent the passage of light, and (ii) 12 h light /dark under continuous irradiation illuminated by 8 fluorescent sources of white light (Sylvania Grolux 35 W). PAR was measured in different positions of the growth chamber (PAR sensor LI-COR-DATALOGGER Inc.USA) at the beginning and the end of the experiments; the mean value was 85 ± 9 μmol m^− 2^ s^− 1^.

Different darkness treatments and thermal regimes on five cattail seed populations (**Ba**, **Cu**, **Ma**, **Se** and **To**) were studied as factors that could alter the final germination and the result of the germination model. The relationship between thermal time, temperature and darkness for the cattail seeds was studied. For this reason, different darkness treatments (0, 3, 5, 7, 10 and 20 dark days) were included in this study. The longest number of days was the same (20 days) for the seeds, so the number of days with 12 h light /dark photoperiod was reduced successively (Table 8). In the treatment DT20d, the seeds were incubated in total darkness for the entire time.

**Table 8:** Thermal regimes and darkness period treatments used in the experiment

Thermal regimes (°C)			Darkness treatments	
Treatments	T_m_	ΔT	Treatments	Description
C 17.5	17.5	0	DT0d	20 days with 12 h light /dark
C 20.0	20	0	DT3d	3 days (24 h darkness) and 17 days (12 h light /dark)
C 22.5	22.5	0	DT5d	5 days (24 h darkness) and 15 days (12 h light /dark)
C 25.0	25	0	DT7d	7 days (24 h darkness) and 13 days (12 h light /dark)
C 27.5	27.5	0	DT10d	10 days (24 h darkness) and 20 days (12 h light /dark)
C 30.0	30	0	DT20d	20 days with 24 h darkness
A 15/20	17.5	5		
A 15/25	20	10		
A 15/30	22.5	15		
A 20/25	22.5	5		
A 20/30	25	10		
A 25/30	27.5	5		

Different thermal regimes were included in this study. These regimes include different levels of constant or alternating temperatures, as explained below. The constant temperatures used (C) were: 17.5, 20.0, 22.5, 25.0, 27.5 and 30.0 °C; the alternating temperatures used (A) were 15/20, 15/25, 15/30, 20/25, 20/30, and 25/30 °C. In the alternating regimes, higher temperatures coincide with light periods and lower temperatures with the dark period of each photoperiodic regime used, and the temperature difference (ΔT) between the dark and light periods was ΔT = 5, 10 and 15 °C (Table 8). The mean value of both temperatures (T_m_) was used to calculate the models.

Temperatures above 30 °C were not used because *T. domingensis* is a plant species whose germination season coincides with middle spring, so it would be very odd if the mean temperature above 30 °C was reached at that time in the study area. The treatment name was composed of the name of the population, mean temperature value, letter of the temperature regime C/A with a number that indicated the ΔT and darkness treatment, such as Ba25C0DT5d and Ba25A10DT3d.

The experimental design was completely randomized. A total of 360 treatments were carried out and three replicates of 33 seeds each were used for each treatment. The germination test was conducted in a filter paper-lined Petri dish filled with 15 ml of distilled water. To prevent evaporation losses, the edges of the Petri dishes were closed using laboratory film. HOBO U12 (Onset Computer Corporation, Pocasset, MA, USA) data loggers were used to monitor the temperature inside the growth chambers. Data from the chamber were accepted if the temperature registered showed a difference of less than ±0.5 °C. All treatments were set up at 9:00 h. Germinated seeds were counted daily for 20 days. A seed was considered to have germinated when the coleoptile broke the pericarp [22].

### Thermal time model

Seed germination of different populations of *T. domingensis* was described as a function of the thermal time model. According to the model presented by Garcia-Huidrobo et al. (1982), the parameter thermal time *θ*_*T*_ (degree-day/ degree-hour) for the percentile g is:

1 $$ {\uptheta}_T(g)=\left(T\hbox{-} {T}_b\right){t}_g $$

Another parameter defined in this model is the germination rate (GR_g_), which is the inverse of the time to radicle emergence of a specific percentile of the population defined by eq. 1.

2 $$ {GR}_g=\frac{T\hbox{-} {T}_b}{\theta_T(g)}=\frac{1}{t_g} $$

There is a linear regression line between GR_g_ and T when the temperature is between T_b_ and T_o_. Under these circumstances, the slope of this linear regression is equal to the reciprocal of thermal time *θ*_*T*_(g). If the change in *θ*_*T*_(g) within a seed population is a log-normal distribution, then the relation between GR_g_ and *θ*_*T*_(g) can be described using the probit function [46].

3 $$ {\mathrm{prob}}_{\mathrm{g}}=\left(\frac{1}{\sigma_{\uptheta_{\mathrm{T}}}}\right)\log \left(\mathrm{T}\hbox{-} {\mathrm{T}}_{\mathrm{b}}\right){\mathrm{t}}_{\mathrm{g}}\hbox{-} \frac{\log \left({\uptheta}_{\mathrm{T}}(50)\right)}{{}^{\sigma_{\uptheta_{\mathrm{T}}}}} $$

In this function, prob_g_ is the probit transformation of the cumulative germination percentile g, *θ*_*T*_(50) is the thermal time to 50% germination, and σ*θ*_*T*_ is the standard deviation of *θ*_*T*_ for individual seeds in the population [70]. Alternative use of probit transformation is logistic transformation when the sample size is not very large. In this case, the midpoint of the logit regression (logit = 0) is the same as that obtained with probit transformation, and the slope (α) is related to the standard deviation of the normal distribution (σ) as defined [71] as:

$$ \upsigma =\frac{\uppi}{\sqrt{3}}\ast \frac{1}{\upalpha} $$

and the log *θ*_*T*_(50) is related to the intercept of the logit regression (β) [27] as:

$$ \log \left({\uptheta}_{\mathrm{T}}(50)\right)=-\frac{\beta }{\alpha } $$

Data from the different temperature regimes were normalized following the concept of thermal time basis (Covell et al., 1986) where *θ*_T_(50) is the mean thermal time to 50% germination used for the log thermal time distribution that was estimated from the equation:

$$ \theta {T}_{(50)}=\frac{\left({T}_m-{T}_b\right)}{GR_{50}} $$

The different variables to solve this equation were obtained as follows:

Equation 2 (1/t _(50)_) was used to calculate GR_50_ for different treatments. In each treatment, the results of GR_50_ were used to calculate a linear regression whose x-intercept represented the estimated value of T_b_ [46, 61]. The T_o_ was obtained from the relationship between different percentiles of GR (30, 50 and 70%) and the T_m_ of each thermal regime (C or A) with the same darkness treatments where T_o_ was the point on the x-axis that coincided with the maximum GR_30_, GR_50,_ and GR_70_ of the above relationship. The estimated θ_T_(50) was used to obtain the σ of the log thermal time in the different treatments and using the logit model [29], the median germination time was estimated using the values to logit = 0 as was mentioned above. Only GR% values less than 95% from T_b_ to T_o_ were included in the logit regression [28]

### Validation of thermal time model

The evaluation process is an important part of the design of a model because it allows checking the concordance between expected and observed results. The method of evaluation used in this work is based on the coefficient of determination (R^2^) and the root-mean-square error (RMSE). This method provides an estimation of the difference between log(*θ*_*T*_(g)) obtained in each model (expected values) and log(*θ*_*T*_(g))_To_ (observed values), where log(θT(g)_To_ was calculated applying thermal parameters of each model (Table 4) to data germination of **To** population.

### Statistical analyses

The multifactor analysis of variance with final germination response as a percentage was carried out with the software package Statgraphics Centurion XVI (Starpoint Technologies, 2011) to determine the relationships among origin, thermal conditions and darkness period of cattail seeds. Germination responses were transformed to meet the assumption of the ANOVA. In this case, the transformation used was arcsine (√final germination %). A multiple range test was also performed to determine which variables were significantly different from the others. The method to make the comparisons was LSD (Least Significance Difference). Statistical differences were defined as *p* < 0.05.

### Availability of data and materials

All data generated or analysed and its supplementary information files during this study are included in this published article. The datasets used and/or analyzed during the current study are available from the corresponding author on reasonable request.

## Supplementary information

**Additional file 1.**

## Data Availability

All data generated or analyzed and its supplementary information files during this study are included in this published article. The datasets used and/or analyzed during the current study are available from the corresponding author on reasonable request.

## Abbreviations

ΔT: The temperature difference
*θ*_*T*_(g): Thermal time
σ*θ*_*T*_: Standard deviation of *θ*_*T*_
A: Alternating
ANOVA: Analysis of variance
Ba: Badajoz
C: Constante
Cu: Cuenca
DT: Darkness treatments
g: the percentile of germination
GA: Agroenergy Group
GFF: Green floating filter
GR: Germination rate
IUCN: International Union for Conservation of Nature and Natural Resources
LD: light/dark
LSD: Least Significance Difference
Ma: Madrid
PAR: Photosynthetic Active Radiation
prob_g_: Probit transformation
Se: Seville
T_b_: Base temperature
T_m_: Mean temperature
t(_g_): time of germination
T_o_: Optimum temperature
To: Toledo
USA: United States of America
